# The elephant in the room

**DOI:** 10.1097/01.NURSE.0000721744.97686.b8

**Published:** 2020-10-20

**Authors:** Deborah C. Roberts

**Affiliations:** **Deborah C. Roberts** is an RN in New York.

**Keywords:** African American nurses, nurses of color, racism, racism in nursing, racial stamina, white fragility

## Abstract

Nurses of color refrain from speaking up when facing racism in their nursing practice from patients, White peers, coworkers, and higher-ups. Racism is the elephant in the room that no one wants to talk about. This article examines the barriers that may prevent nurses of color from speaking openly about racism and encourages all nurses to speak up and out against racism in nursing.

## Abstract 2

Nurses of color refrain from speaking up about racism they experience from patients, White peers, coworkers, and higher-ups. Racism is the elephant in the room that no one wants to talk about. This article examines the barriers that may prevent nurses of color from speaking openly about racism and encourages all nurses to speak up and out against racism in nursing.

**Figure:**
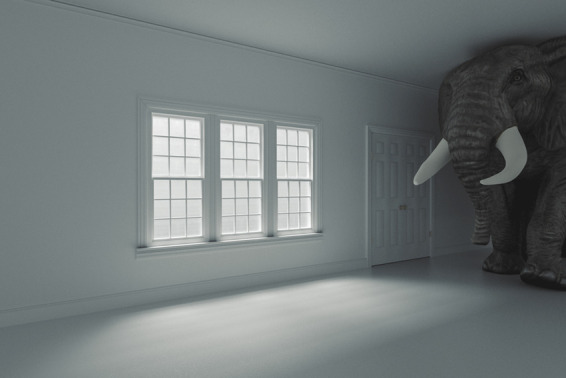
No caption available.

MY EARLIEST childhood memories are not of the turbulent civil rights struggles of the 1960s, but of Mr. Neary,∗ my mother, and me. Mr. Neary, the principal of my elementary school, could always be found sitting behind his imposing gray metal desk. Trailing my mother into his office, I would take my place at her side. She had come to talk.

We were one of the first African American families to integrate a small New England town. The neighbors—and their children— made it clear they objected to our presence. Realtors repeatedly knocked on our door to entice us with lucrative offers to move. Each day at school I was subjected to name-calling and bullying, with an occasional beating thrown in for good measure. My mother, never afraid to speak her mind, frequently visited Mr. Neary to address these issues as well as the poor academic curriculum laid out for me.

My eyes darted back and forth between them as they talked. Nonchalantly, Mr. Neary said, “I am sorry, but Negros are only cut out to be cooks and maids.” My courageous mother, standing tall and erect, looked squarely at Mr. Neary and firmly responded that racism and discrimination were unjust, and she wasn't having any of it.

Mr. Neary's body stiffened. His face registered displeasure slowly morphing into outright anger. These visits to Mr. Neary's office taught me two valuable lessons: (1) discussing prejudice and racism makes people very uncomfortable, and (2) it takes courage to talk about it.

I am an African American RN. Over the years I have listened to countless stories from nurses of color recounting their experiences facing prejudice and racism from patients, White peers, coworkers, and higher-ups. Usually before telling their story they glance nervously around the room to ensure our conversation is not overheard. Their voices drop as they begin to whisper their story and whisper their pain. I encourage them to speak up. Most responses share a common denominator—they lament the futility of using their voice, fearing personal and professional consequences for speaking their truth. As one coworker put it, “the racism we deal with as nurses of color is the elephant in the room that no one wants to talk about.”

Let's talk about it.

In the United States of America, racism is a problem. Therefore, racism in nursing in the United States of America is also a problem.

There it is—I said it.

The World Health Organization crowned 2020 the International Year of the Nurse and the Midwife.^1^ But 2020 is also the year COVID-19 reached our shores and swept the nation. Fear and panic took over. Breonna Taylor...Warnings flashed – Stay Inside to Stay Alive! PPE, contact tracing, quarantine, and social distancing! Ahmaud Arbery...Heart-wrenching sights, sounds and images of the courage and heroism of nurses and other healthcare professionals were on full display. George Floyd. Long ago the fuse on the powder keg was lit and now the world had a front row seat to watch it explode. Videos of Mr. Floyd's murder began saturating the airways. Across the nation and around the world, people of all colors flung open their doors and flooded the streets in protest. Doctors, nurses, and other healthcare workers walked off the job, to kneel side by side in a show of solidarity to protest racism and the killing of George Floyd. Demands to acknowledge and end racism and injustice faced by people of color have been placed on the table—and they are not going away.

## What is racism?

Acosta and Ackerman-Barger describe three pillars of racism:^2^

- personally mediated racism (based on prejudice, whether intentional or unintentional)
- internalized racism (accepting biases and stereotypes attributed to a group)
- institutional racism (using power in recognition and support of these norms to perpetuate inequities that favor one group over another).

If racism is a problem for all people of color, why discuss racism in nursing? According to the Bureau of Labor Statistics, as of 2019, almost 90% of RNs are women. Of these, 76% are White, 12% are Black/African American, and 12% are Latino/Asian.^3^ Assuming that most Black nurses are women, racism in nursing directly and negatively impacts this subset of nurses—women whose struggles have historically been overlooked and undocumented.

As licensed professionals, nurses of color face unique challenges when contending with racism that differ greatly from those faced by other healthcare professionals of color, such as physicians. Many of these nurses, fully aware of the risks involved in raising their voice and fearing backlash, make a conscious decision to lower their voice to a whisper. Why?

***Nurses of color refrain from speaking up and out against racism because many of their White coworkers fail to understand the implications of racism in the lives of people of color***.

Soon after COVID-19 began to touch our lives, my coworkers and I made a habit of regularly connecting with each other via group messaging. TikToks, emojis, and poems flowed back and forth on our phones—anything to keep us smiling, laughing, and feeling connected.

Shortly after the murder of George Floyd, a White coworker forwarded a video of a woman of color commenting that people of color should wake up, realize that George Floyd had a sordid past, and stop making a fuss over him—he was no angel. A few days later, the same coworker forwarded another video of an African American man saying that Black people should stop blaming White people for all our ills. He issued an apology to White people on behalf of the entire Black race. He went on to say that White people should feel no need to kneel in protest and solidarity because they bear no responsibility for the sufferings of African Americans.

Staring at the screen, I wondered less about the content of the video than of the freeness with which my White colleague forwarded it. I thought of the numerous times I have been shadowed by store security watching my every move. I recalled instances when I was pulled over by police officers who approached my car with their hands ever so gently, but firmly, on their guns. I thought of the conversation that every Black mother and father *must* have with their sons (and daughters) about how to survive an encounter with armed law enforcement—a conversation that my White coworker will never have or understand the need to have with her sons. More important, my White coworker will never share the pain of the mothers of Laquan McDonald, Tamir Rice, or Trayvon Martin.

I hear a ping on my cell phone. Looking down I see one nurse of color leave the group, then another.

Efforts to combat racism in nursing will continue to be frustrated as long as a deep divide in the perceptions of racism and its impact in the lives of people of color persists between people of color and White people. In the words of James Baldwin, “not everything that is faced can be changed, but nothing can be changed until it is faced.”^4^

***Nurses of color do not engage in cross-racial discussions with their White coworkers because speaking about racism often triggers an overwhelming stress response in White coworkers who lack the racial stamina to cope***.

Sitting in the break room, my coworkers and I intently watched as a breaking news story of racial violence against a man of color flashed across the TV screen. I commented on the story and began recounting a childhood experience when I was violently touched by racism. A White male coworker became angry, jumped out of his chair, and yelled, “that did not happen to you! Do you know the discrimination I face and how hard it is for me as a White man?!” Visibly upset and angry, he stormed out of the room. A coworker followed, reappeared, and exclaimed “I think he is headed to Human Resources!”

Robin DiAngelo is a White racial justice educator who defines White people's reactions to discussions about race and racism as “white fragility.”^5^ She offers a unique perspective to “speak to the White experience” from the inside looking out, contending that most White people refuse to admit their racism, or the impact of being socialized in a white supremacist culture created, sustained, and perpetuated by White people to their advantage and to the detriment of people of color. Ultimately, because many White people see themselves as individuals and refuse to see themselves in racial terms, they are unwilling to concede they are the direct beneficiaries of racism or play any role in perpetuating it.^5^

DiAngelo observed recurring patterns of emotions and reactions among many White people when engaging in discussions about racism. White participants would begin to display emotions such as defensiveness, anger, withdrawal, hostility, crying, or distress in discussions about race. As overwhelming racial stress took over, white fragility caused participants to react in various predictable ways, such as claiming they were the victim of reverse discrimination, accusing the facilitator of playing “the race card,” or saying they felt threatened. In some cases, DiAngelo noted that when a person of color pointed out to a White person the impact of behavior perceived as racist, the White person sought out another person of color to collude with in order to discredit and invalidate the claim of racism. Ultimately, white fragility empowers White participants to end cross-racial discussions, regain their racial comfort, and maintain their dominant status in the racial hierarchy.^5^

Despite the current racial climate, many nurses of color continue whispering that they fear the consequences of speaking out against the elephant in the room. Undeniably, those who dare to courageously speak out risk backlash in their nursing practice. Maybe we lower our voice fearing that too much is at stake to risk losing our livelihood and the ability to care for our families. Why?

During the 400 years that have elapsed since enslaved people were first brought to America, the expectation for people of color has not changed. The expectation was and continues to be that we suppress our feelings and deny the pain inflicted by racism. After hundreds of years during which our very survival depended on whispering, we have become skilled at it. I must admit that even though I work hard to imitate my mother's courageous voice, at times I too feared the consequences of speaking out and lowered my voice to a whisper.

## Call out the elephant in the room

Undeniably, each nurse of color will make a personal decision about how to respond to the racism they face in their nursing practice. To realize meaningful, substantial change, both Black and White nurses must do their part to oust the elephant from the room. How? First, White nurses must develop the racial stamina to hear our story and engage in authentic cross-racial discussions. Second, nurses of color must speak up concerning the toll that surviving racism exacts in our lives. We cannot ask our White peers to admit to, challenge, and overcome their racism if we refuse to openly acknowledge its existence and impact in our lives. Our hope is that one day our White fellow nurses will possess the racial stamina to sit in stillness, listen to our story, and hear our pain. But whether they continue adrift on the course established hundreds of years ago by their forefathers or choose to raise their sails, our course must not change. We cannot maintain racial silence. We must summon the courage to tell our story.

Each time my mother marched into my principal's office she taught me a valuable lesson—the need for courage to speak out against what is wrong, stand up for what is right, and the life-changing impact that using our voice can have on others. True, there are consequences for speaking up, but more important, there are consequences for remaining silent. As George Yancy says, “it is not always what you say that does the harm, it is also what you don't say, what you fail to say, refuse to say.”^6^ Turning away, feigning ignorance and remaining silent has implications for us all.

## References

[1] World Health Organization. WHO campaigns. Year of the nurse and the midwife 2020. www.who.int/campaigns/year-of-the-nurse-and-the-midwife-2020.

[2] Acosta D Ackerman-Barger K. Breaking the silence: time to talk about race and racism. *Acad Med*. 2017;92(3):285–288. 27655050 10.1097/ACM.0000000000001416

[3] US Bureau of Labor Statistics. Labor force statistics from the current population survey. 2020. www.bls.gov/cps/cpsaat11.htm.

[4] Wilson DE *Treasury of Black Quotations*. Lincoln, NE: Authors Choice Press; 2004.

[5] DiAngelo R *White Fragility: Why It's So Hard for White People to Talk About Racism*. Boston, MA: Beacon Press; 2018.

[6] Yancy G *Backlash: What Happens When We Talk Honestly About Racism in America*. Lanham, MD: Rowman & Littlefield; 2018.

